# Increased fluoroquinolone resistance with time in *Escherichia coli *from >17,000 patients at a large county hospital as a function of culture site, age, sex, and location

**DOI:** 10.1186/1471-2334-8-4

**Published:** 2008-01-15

**Authors:** Lauren Becnel Boyd, Robert L Atmar, Graham L Randall, Richard J Hamill, David Steffen, Lynn Zechiedrich

**Affiliations:** 1 Department of Molecular Virology and Microbiology, Baylor College of Medicine, Houston, TX, 77030, USA; 2 Department of Medicine, Baylor College of Medicine, Houston, TX, 77030, USA; 3 Interinstitutional Program in Structural and Computational Biology and Molecular Biophysics, Baylor College of Medicine, Houston, TX, 77030, USA; 4 Bioinformatics Resource Center, Baylor College of Medicine, Houston, TX, 77030, USA; 5 Michael E. DeBakey Veterans Administration Medical Center, Houston, TX, 77030, USA

## Abstract

**Background:**

*Escherichia coli *infections are common and often treated with fluoroquinolones. Fluoroquinolone resistance is of worldwide importance and is monitored by national and international surveillance networks. In this study, we analyzed the effects of time, culture site, and patient age, sex, and location on fluoroquinolone resistance in *E. coli *clinical isolates.

**Methods:**

To understand how patient factors and time influenced fluoroquinolone resistance and to determine how well data from surveillance networks predict trends at Ben Taub General Hospital in Houston, TX, we used Perl to parse and MySQL to house data from antibiograms (n ≅ 21,000) for *E. coli *isolated between 1999 to 2004 using Chi Square, Bonferroni, and Multiple Linear Regression methods.

**Results:**

Fluoroquinolone resistance (i) increased with time; (ii) exceeded national averages by 2- to 4-fold; (iii) was higher in males than females, largely because of urinary isolates from male outpatients; (iv) increased with patient age; (v) was 3% in pediatric patients; (vi) was higher in hospitalized patients than outpatients; (vii) was higher in sputum samples, particularly from inpatients, than all other culture sites, including blood and urine, regardless of patient location; and (viii) was lowest in genital isolates than all other culture sites. Additionally, the data suggest that, with regard to susceptibility or resistance by the Dade Behring MicroScan system, a single fluoroquinolone suffices as a "surrogate marker" for all of the fluoroquinolone tested.

**Conclusion:**

Large surveillance programs often did not predict *E. coli *fluoroquinolone resistance trends at a large, urban hospital with a largely indigent, ethnically diverse patient population or its affiliated community clinics.

## Background

*E. coli *is the most common etiologic agent of infections caused by Gram-negative bacilli, and these infections routinely are treated with fluoroquinolones, some of the most-frequently prescribed antibiotic classes [1]. National and international surveillance networks track the frequency of susceptibility to antimicrobial agents, including the fluoroquinolones. Some fluoroquinolone data, such as that showing that males are more likely than females to have resistant isolates, reveal clear trends [2-4]. Other data from these networks can vary. For example, one study uncovered that younger patient age was associated with increased likelihood of having a ciprofloxacin non-susceptible isolate [4], but another report of urinary *E. coli *isolates found that resistance was highest in patients ≥ 65 years of age [5]. Low numbers of isolates from each participating hospital, variations in patient populations, and differences in geographical regions of these hospitals may play a role in the variation in the data. Large-scale, local studies, therefore, are required to understand drug resistance in a given community.

## Methods

We analyzed the effects of patient factors on fluoroquinolone resistance over time at Ben Taub General Hospital, a 578 bed, acute-care, county hospital that serves a mostly Hispanic and African-American patient population in Houston, Texas. The hospital microbiology laboratory also provides service to twelve community health centers across Harris County, Texas. This retrospective study differs from surveillance network studies in that data from thousands of *E. coli *isolates from a single hospital laboratory were analyzed simultaneously. Antibiotic susceptibilities were determined using the Dade Behring MicroScan system (Sacramento, CA, USA) according to Clinical Laboratory Standards Institute (CLSI) guidelines [6]. Data from all *E. coli *antibiograms from July 1, 1999 to December 31, 2004 (n ≅ 21,000) were parsed with Perl and imported into a MySQL database (Uppsala, Sweden). All database queries only included information from the first isolate for each patient (n ≅ 17,000). Female patients outnumbered males 3.5 to 1, with age ranging from 0.01 to 103 years (average females = 39.5 ± 20.6, males = 41.9 ± 24.9). Ciprofloxacin, gatifloxacin, levofloxacin, norfloxacin, and ofloxacin were included among the ~25 different antibiotics in the antibiograms, although gatifloxacin has since lost approval for systemic, but not ophthalmic, use. Chi square analysis and the Bonferroni correction were used to analyze all the data, and *P *≤ 0.01 (99% confidence interval) was required for statistical significance. We also determined the odds ratio of resistance to susceptibility with a multiple logistic regression equation using SPSS (Chicago, IL, USA).

## Results and Discussion

### Fluoroquinolone resistance increased with time

**Figure 1:**
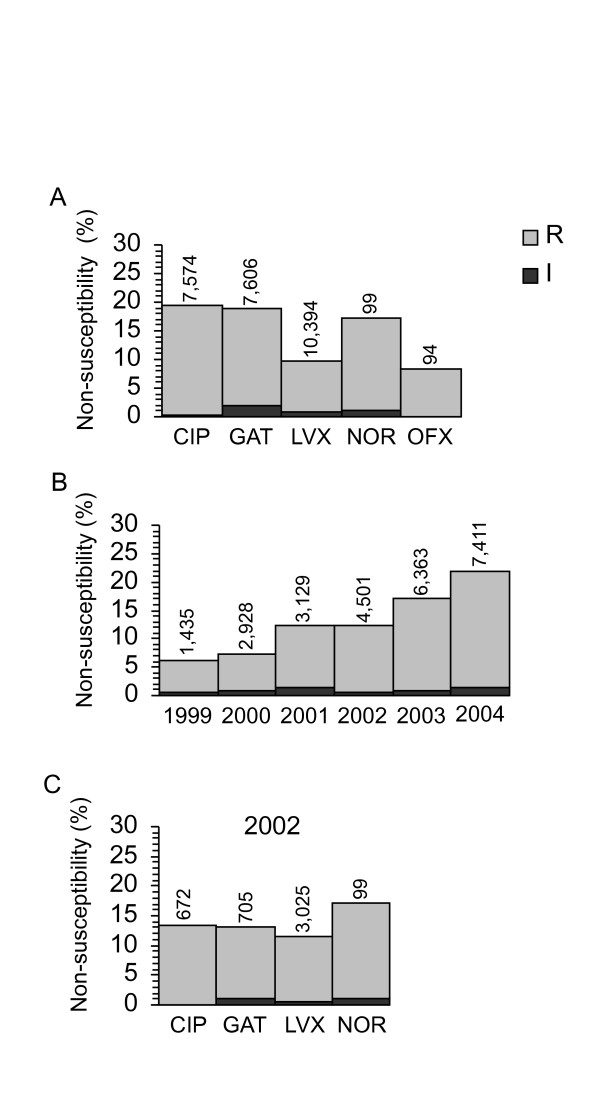
**Fluoroquinolone resistance**. The following fluoroquinolone abbreviations were used in this and subsequent figures: CIP, ciprofloxacin; GAT, gatifloxacin; LVX, levofloxacin; NOR, norfloxacin; and OFX, ofloxacin. For these and subsequent graphs, the number of isolates in each category is shown above each bar. The frequency of resistance increased significantly by Chi-square tests (*P *≤ 0.01) each year except from 1999 to 2000 and 2001 to 2002. (A) Average percentage of non-susceptible isolates from 1999 to 2004 for each fluoroquinolone. Resistant ("R") and intermediate ("I") isolates for each fluoroquinolone are shown. With regards to statistical significance, levofloxacin and ofloxacin resistance was the same by Chi-square analysis, and both were distinct from ciprofloxacin, gatifloxacin, and norfloxacin. (B) Total fluoroquinolone non-susceptibility over time. The average percentage of "R" and "I" isolates for all the tested fluoroquinolones combined for each year is shown. (C) Percentage of non-susceptible isolates for each fluoroquinolone in 2002.

Surveillance networks reported that ~5% of United States isolates were fluoroquinolone resistant [2-4,7]. Data averaged from 1999 – 2004 for each fluoroquinolone, however, showed that resistance exceeded the previously reported values by 2- to 4-fold (Fig. 1A). As expected (because ofloxacin is a racemic mixture of levofloxacin and its inactive enantiomer), the prevalence of levofloxacin and ofloxacin non-susceptibility was nearly identical (~10%). The frequency of non-susceptibility to levofloxacin and ofloxacin was approximately half that of ciprofloxacin, gatifloxacin, or norfloxacin (~18%), and the latter three were statistically indistinguishable (Fig. 1A). Regardless of year or drug, < 2% of isolates were intermediate resistant (Figs. 1A, 1B, and 1C); data from intermediate resistant isolates were not included in subsequent analyses. When data from all fluoroquinolones tested in a given year were combined, the frequency of non-susceptibility increased with time from ~6% to almost 25% (Fig. 1B). Binary regression showed that, with each passing month, the odds of having a resistant isolate significantly increased 1.024-fold (*P *< 0.001). Two other studies from the region found a similar high resistance frequency. One found ~13% of *E. coli *from febrile, neutropenic cancer patients undergoing chemotherapy at Houston's M.D. Anderson Cancer Center were ciprofloxacin resistant [8]. Fluoroquinolone prophylaxis is common in these patients, so one might expect a higher incidence of resistance than in the general population. The second found that ~20% of the 59 isolates from the urine of outpatients in the "West South Central" (Arkansas, Louisiana, Oklahoma, and Texas) area were resistant to levofloxacin and ciprofloxacin [5]. Thus, it may be that fluoroquinolone resistance is significantly higher for patients in Texas than the rest of the nation.

It has been thought that fluoroquinolones exemplify a "class effect," such that when resistance mechanisms decrease susceptibility for one drug, they do so simultaneously for all [9]. Each mechanism, however, affects different fluoroquinolones to varying extents [10], and some mechanisms, such as Aac(6')-Ib-cr [11] and QepA [12], decrease susceptibility only to ciprofloxacin and norfloxacin. Thus, it was important to distinguish whether time or specific fluoroquinolone accounted for the resistance differences shown in Fig. 1A. Comparing fluoroquinolones for a single year (2002), in which ciprofloxacin, gatifloxacin, and levofloxacin were all tested routinely, revealed that the frequency of resistance for the three fluoroquinolones was statistically indistinguishable, ~15% (Fig. 1C). An independent test of whether there were any variations among the fluoroquinolones was to look for isolates that were "I" or "R" to one drug and "S" to another. When ciprofloxacin and gatifloxacin (n = 6,272), levofloxacin and ciprofloxacin (n = 560), or levofloxacin and gatifloxacin (n = 582) were analyzed, 1–4% of isolates were non-susceptible to one drug and susceptible to the other. The small number of differentially susceptible isolates were equally likely to be non-susceptible to one drug while susceptible to another, and these few measurement differences likely represent the Dade MicroScan error rate [13].

To test whether time or specific fluoroquinolone accounted for the differences in Fig. 1A, we measured fluoroquinolone MICs by the agar dilution method independently in our laboratory [6]. We quantified MICs in 242 representative isolates from Ben Taub that were collected over the duration of this study. When ciprofloxacin, gatifloxacin, levofloxacin, and norfloxacin MICs were compared, all isolates that had non-susceptible MICs to one fluoroquinolone had non-susceptible MICs for all four drugs. Thus, what initially appeared to be drug differences in Fig. 1A were, in fact, a consequence of which drugs were tested over time. Our data and others [14] suggest that the susceptibility status of at least ciprofloxacin, gatifloxacin, levofloxacin, and norfloxacin could be inferred from testing only one of these drugs as a "surrogate marker." Because of these data, we conclude that fluoroquinolones indeed exhibit a class effect with regard to susceptibility as measured by the Dade Behring MicroScan system, and we combine data for all fluoroquinolones in subsequent analyses.

### Variation in resistance in the hospital and at outpatient community clinics

Unlike most antibiotics in the United States, ciprofloxacin resistance has not been reported to be higher in isolates from patients in the ICU than in inpatients and outpatients [3,4,15]. In our population, however, resistance (~19% of >4,000 patients) in ICU and inpatient isolates (statistically indistinguishable from each other) occurred more frequently than in outpatients (~9% of >12,500 patients, *P *< 0.0001; data not shown). Hospital outpatients were significantly more likely (*P *< 0.001) to have a resistant isolate (11%) than outpatients who received care from the twelve community health centers (~8%). Resistance at most community clinics was statistically indistinguishable from community outpatients as a whole, except for two clinics with significantly higher resistance (~14%, *P *< 0.01) and two other clinics where resistance was significantly lower (~3%, *P *< 0.001). Outpatient community health centers serviced by the hospital microbiology laboratory provided care to all patients seeking treatment, with the exception of the clinic with the highest prevalence of resistance, which provides care for HIV-positive patients. Unlike the HIV clinic, patients at all other clinics did not have any known specific illness. These data show that the majority of outpatients had the same likelihood of having a resistant isolate, regardless of whether they received care in the community or at the hospital.

### Fluoroquinolone resistance as a function of culture site

Isolates from abscesses, bloodstream, exudate, fluids and wounds were not different from each other or from urine (~10% resistant, Table 1). Genital isolates (all from females) were significantly less likely (0.1%, *P *< 0.0001) to be resistant than isolates from other sites. Sputum samples had the highest frequency (~30%) of fluoroquinolone resistance (*P *= 0.001), with resistance being significantly more likely in inpatients (~40%) than ICU (~20%) patients. Chronic bronchitis, pneumonia, or complications of emphysema (all of which are common in chronic obstructive pulmonary disease, or COPD, patients) are often treated with fluoroquinolones, which facilitate recovery in hospitalized patients [16,17]. Thus, it is possible that the resistant sputum isolates originated from patients suffering from COPD. Miscellaneous other culture sites (Table 1) were too infrequent to be included in statistical analyses; however, bone (~50% resistant), bronchia (~30%), and catheter tip (~40%) isolates also had alarmingly high frequencies of resistance (Table 1).

**Table 1:** Isolate culture sites as a function of susceptibility status

		**Number of isolates**		
		
	**Culture Site**	**S**	**I**	**R (%)**
				
Included^a^	Abscess	388	7	51 (11)
	Blood	864	16	175 (17)
	Exudate	602	2	84 (12)
	Fluids	273	2	36 (12)
	Genitals	259	1	2 (1)
	Sputum	271	4	128 (32)
	Urine	12,382	197	1,461 (11)
	Wounds	237	1	32 (12)
Not included	Bone	4	0	5 (56)
	Bone marrow	2	0	0 (0)
	Bronchia	13	0	6 (32)
	Catheter tip	38	3	24 (37)
	Cerebrospinal fluid	12	0	0 (0)
	Ear	7	0	0 (0)
	Enteric	2	0	0 (0)
	Eye	26	0	5 (16)
	Miscellaneous Tissue	37	0	9 (20)
	Respiratory Tract	21	0	3 (12)
	Trachea	33	0	4 (11)

^a^Subjected to statistical analyses. Culture sites with fewer than five "S," "I," or "R" isolates were not analyzed because of insufficient statistical power.

### Fluoroquinolone resistance as a function of patient age

**Figure 2:**
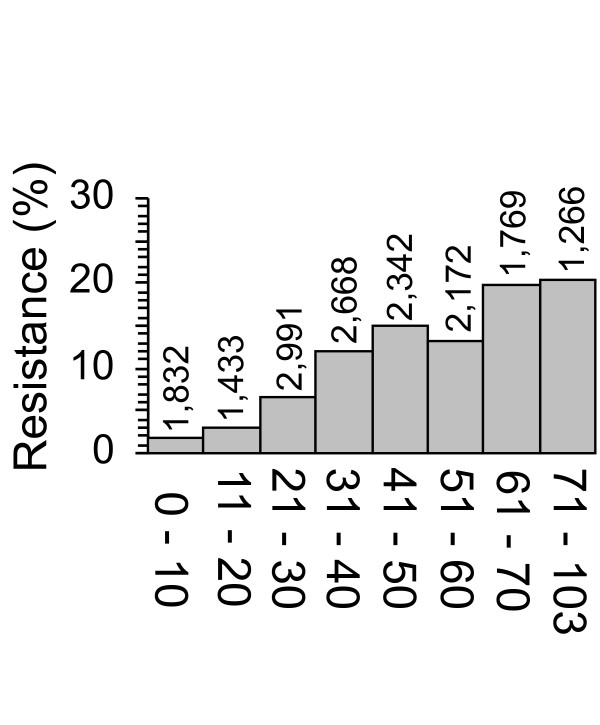
**Fluoroquinolone resistance as a function of patient age**. The percentage of resistant isolates is shown by age bracket. Patients ≥ 70 years old were grouped. All data were analyzed by binary regression and Chi-square tests. With regards to statistical significance, overall, fluoroquinolone resistance increased with patient age.

We determined the percentage of isolates that were fluoroquinolone resistant in each 10-year age bracket up to 70 years of age. Because relatively few patients were older than 70 years, patients of age 71 – 103 were grouped together. We found, overall, that fluoroquinolone resistance increased significantly with patient age (Fig. 2; *P *< 0.001), and that the odds of an isolate being resistant increased 1.027-fold for each year of age (*P *< 0.0001). The increasing resistance with patient age was not associated with a specific patient location. Because decreased immune function and overall health are associated with advanced age, the increased occurrence of resistance in isolates from older patients may have resulted from more frequent fluoroquinolone exposure than that for younger patients.

Approximately 2% of ≤ 10 year-old children had fluoroquinolone-resistant *E. coli *isolates. These children should have been naïve to fluoroquinolones, as the use of these antibiotics in pediatric patients generally is not recommended. To ascertain whether these children presented with the infections around the same time, which could indicate an outbreak or clonal spread, we determined when and where they had been treated. The majority of children with resistant isolates were outpatients, but no obvious temporal association was detected. Although the only licensed use of fluoroquinolones in children is treatment of post-inhalation anthrax exposure with ciprofloxacin, some compassionate use occurs in children suffering from serious infections, such as multidrug resistant infections [18]. In Houston, children with serious illness generally would be treated at Texas Children's Hospital, not Ben Taub General Hospital. Thus, most pediatric patients at Ben Taub, just as in the United States as a whole, should not have had prior fluoroquinolone exposure. Although our data did not control for previous antibiotic therapy, two previous studies that did also found that ~2% of children harbored quinolone- or fluoroquinolone-resistant isolates [19,20]. Therefore, a small percentage of children may carry fluoroquinolone-resistant *E. coli *independently of prior fluoroquinolone use. The affected children may have acquired the fluoroquinolone-resistant isolates from a family member or in the community, although outbreaks of gram-negative bacteria are rarely reported [21]. However, because such surveillance efforts currently monitor the spread of virulent bacteria like *E. coli *O157:H7, less virulent strains that do not cause significant morbidity might be passed undetected from person to person.

### Increased resistance in urinary isolates from male outpatients

**Figure 3:**
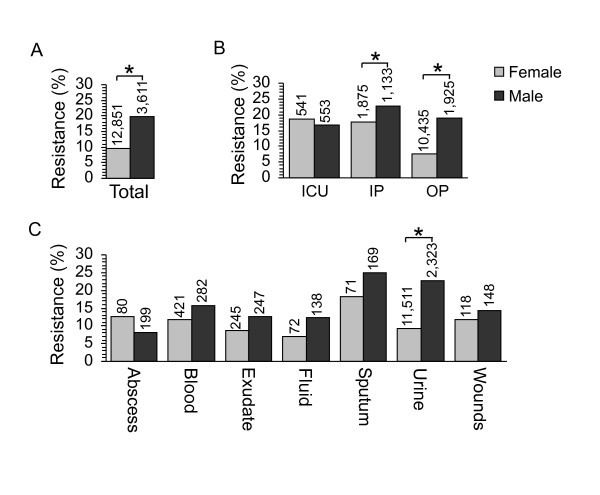
**Fluoroquinolone resistance as a function of patient sex**. Asterisks denote statistically significant differences. All data were analyzed by binary regression and Chi-square tests. (A) The percentage of resistant isolates is shown for female and male patients. (B) Fluoroquinolone resistance in female and male patients as a function of location. Because so few fluoroquinolone-resistant genital isolates existed, these data were excluded from analysis. (C) Fluoroquinolone resistance in female and male patients as a function of culture site.

Like previous studies [3,4,22,23], males from Ben Taub between 1999 to 2004 were significantly more likely to have resistant isolates than females (Fig. 3A). We addressed whether patient location or culture site played a role. The percentage of male outpatients with resistant isolates was significantly higher (*P *< 0.001) than female outpatients (Fig. 3B). The sex difference was smaller, but still significant (*P *= 0.001) for inpatients, and no resistance difference between the sexes existed in isolates from ICU patients. Although for the majority of culture sites the sexes were indistinguishable, the frequency of resistance was significantly higher (*P *< 0.001) in urine cultures from males compared to females (Fig. 3C). Increased fluoroquinolone resistance in males, therefore, is attributable to isolates from the urine of outpatients. Unlike female urinary tract infections (UTIs), male UTIs are frequently complicated and are more likely to require prolonged antimicrobial therapy, potentially explaining the fluoroquinolone resistance discrepancies between the sexes [24,25]. Thus, differences in the type of UTI may impact the variation in resistance between the sexes. Our data cannot distinguish males with urinary tract infections from those with prostatitis. Fluoroquinolones are used to treat chronic prostatitis, even though they do not all readily penetrate the prostate [26,27]. Doses of fluoroquinolones that are less than the minimal inhibitory concentration (MIC) lead to selection of resistance mutations by a process known as "sub-inhibitory MIC effects" [28,29]. It is possible that such selection could play a role in the difference in resistance frequency between the sexes.

## Conclusion

Fluoroquinolone resistance patterns are complicated, making it difficult to apply national data to clinical practice in any specific area. Studies that identify resistance trends on a local or regional scale ([30,31], this study) are more directly applicable to a given area and could help better guide prescribing practices of clinicians. In addition, it is important to disseminate such regional data, as they might be harbingers of trends that may spread to or be encountered in other regions in the future. This is particularly true for the fluoroquinolones, because their prescribing increased sharply since the 1990s in the United States [32] and likely in other countries worldwide as well. Finally, meta-analyses of regional studies might explain the variations in resistance trends from international surveillance networks and even predict such trends not only for *E. coli*, but also for other bacterial species, given the high conservation of the fluoroquinolone target topoisomerases across divergent species.

## Competing interests

The author(s) declare that they have no competing interests.

## Authors' contributions

LBB parsed the original antibiogram data using Perl, designed and implemented the MySQL database, performed statistical analyses, and wrote the manuscript. RLA obtained the antibiogram data and participated in the data analysis and editing of the manuscript. GLR helped with data analysis, performed binary regression statistical analysis, and edited the manuscript. RJH, DS, and LZ collaborated in the design, data interpretation, and editing of the manuscript. All authors read and approved the final manuscript.

## Pre-publication history

The pre-publication history for this paper can be accessed here:


